# Indications for marker clip in the setting of neoadjuvant/neoendocrine therapy

**DOI:** 10.1186/bcr3309

**Published:** 2012-11-09

**Authors:** ME Fletcher, BJG Dall, N Sharma

**Affiliations:** 1 Leeds Teaching Hospital NHS Trust, Leeds, UK

## Introduction

We are aware that the use of neoadjuvant therapy (NACT) and neoendocrine therapy (NAET) is steadily increasing and more patients are being referred for marker clip placement. This is our experience of the indications for marker clip placement in a large cancer unit using both NACT and NAET for treatment of breast cancers.

## Methods

An 18-month retrospective audit was performed to see which patients were having marker clip placement. We recorded the indication for marker clip, final surgery and whether the marker clip was used for localisation.

## Results

Fifty patients of the 92 had marker clip placement, of which 37 had NACT and 13 NAET. The marker clip was placed in 35/50 as radiology raised the possibility of near or complete pathological response. In 8/50 the marker clip was placed to aid the pathologist because of concern regarding residual low-volume disease in patients having WLE or mastectomy and 7/50 were trial patients. Thirty-eight patients had WLE and the marker clip was localised in 6/38 cases. Ten patients had mastectomy and in two cases no surgery was performed due to proven metastatic disease. Nine patients had complete pathological response.

## Conclusion

Due to NACT and NAET, radiologists and pathologists are facing new challenges in localising and identifying residual low-volume disease following completion of treatment. Marker clip placement can play a crucial role in ensuring accurate localisation at the time of surgery and can aid identification of residual disease for the pathologist.

